# The transcription factor crem-αlpha regulates inflammatory T cell subsets in juvenile idiopathic arthritis

**DOI:** 10.1186/1546-0096-12-S1-P15

**Published:** 2014-09-17

**Authors:** Kim Ohl, Helge Nickel, Halima Moncrieffe, Lucy R Wedderburn, Klaus Tenbrock

**Affiliations:** 1 Deptarment of Pediatrics, Uniklinik RWTH Aachen, Aachen, Germany; 2 Division of Rheumatology, Cincinnati Children’s Hospital Medical Center, Ohio, USA; 3 Infection, Inflammation and Rheumatology Section, Institute of Child Health, UCL, London, UK

## Introduction

The cAMP response element (CRE) modulator (CREM)α binds to promoters of genes with CREs and regulates transcription via a chromatin-dependent mechanism. CREMα is important for the T cell pathophysiology of SLE by suppression of IL-2 and CD3ζ but enhancement of IL-17 transcription. Juvenile idiopathic arthritis (JIA) is an autoimmune disease of unknown origin. Th17 cells have a pathogenic role in arthritis and are not controlled by local FoxP3^+^ regulatory T cells (Tregs). Pathogenic T cells in the inflamed joints of JIA patients have enhanced expression of IL-17, IFN-γ and CD161. CD161^+^ CD4^+^ cells also contain FoxP3^+^ cells that produce proinflammatory cytokines. A higher frequency of CD161 Tregs appears to associate with more severe disease in JIA, a fact which might contribute to the failure by Treg to suppress ongoing inflammation.

## Objectives

The aim of this study was to evaluate the role of CREM-expressing T cells in juvenile idiopathic arthritis.

## Methods

T cells and peripheral blood mononuclear cells (PBMCs) from healthy donors and JIA patients as well as synovial fluid mononuclear cells (SFMCs) from JIA patients were stimulated in vitro with anti-CD3/CD28 antibodies. PBMCS from healthy donors were incubated in the presence of synovial fluid from JIA patients. CREMα was knocked down in PBMCs and SFMCs by transfection with CREM siRNA.Flow cytometry was used to measure CREM protein, CD161, Helios and FoxP3 expression as well as secretion of cytokines. RNA was quantified by quantitative Real-time PCR.

## Results

We observed enhanced expression of CREM in synovial fluid T cells from JIA patients. Enhanced expression of CREM was also induced after ex vivo culture of PBMCs from healthy donors with synovial fluid from JIA patients. We furthermore found enhanced expression of CREM in CD4^+^CD161^+^ and in CD4^+^FoxP3^+^CD161^+^ cells, which are known producers of inflammatory cytokines, compared to CD4^+^CD161^-^ and CD4^+^FoxP3^+^CD161^-^ cells. Vice versa Helios^+^FoxP3^+^ cells, which are supposed to be stable Tregs and do not express inflammatory cytokines, showed lower levels of CREM expression. Incubation with synovial fluid induced the expression of IFN-γ, IL-17 and FoxP3 in T cells, which could be reserved by transfection of CREM siRNA. Within the FoxP3^+^ population, CREMsiRNA reduced CD161^+^FoxP3^+^ cells but hardly affected Helios^+^FoxP3^+^ cells. We next asked what drives enhanced expression of CREM in synovial fluid stimulated T cells and if pharmacologic inhibition of these factors might reduce occurrence of inflammatory Tregs and effetor T cells. Interestingly in in vitro assays both Anakinra as well as Enbrel, which antagonize IL-1 respectively TNF-a signaling downregulated expression of inflammatory cytokines in T cells and both downregulated CREM expression as well. We then aimed to directly block CREM activating by inhibiting Calcium/calmodulin-dependent protein kinase type IV (CaMKIV).We have shown before that SLE serum IgG activates CaMKIV and identified CaMKIV as being responsible for the increased expression of CREM in SLE T cells. Inhibition of CaMKIV in PBMCS markedly downregulated CREM expression and reduced numbers of IL-17 and IFN-γ producing T cells.

## Conclusion

We thus suggest that the overexpression of CREMα in T cells contributes to T cell pathophysiology in JIA by regulating percentages of inflammatory CD4^+^ IL-17^+^ producing cells, as well as inflammatory CD161^+^FoxP3^+^ cells.

## Disclosure of interest

None declared.

